# Academic Primer Series: Key Papers About Teaching with Technology

**DOI:** 10.5811/westjem.2017.2.33076

**Published:** 2017-05-01

**Authors:** Megan Boysen-Osborn, Robert Cooney, Michael Gottlieb, Teresa M. Chan, Aaron Brown, Andrew King, Adam Tobias, Brent Thoma

**Affiliations:** * University of California, Irvine Medical Center, Department of Emergency Medicine, Orange, California; † Geisinger Medical Center, Department of Emergency Medicine, Danville, Pennsylvania; ‡ Rush University Medical Center, Department of Emergency Medicine, Chicago, Illinois; § McMaster University, Department of Medicine, Division of Emergency Medicine, Hamilton, Ontario, Canada; ¶ University of Pittsburgh School of Medicine, Department of Emergency Medicine, Pittsburgh, Pennsylvania; || The Ohio State University Wexner Medical Center, Department of Emergency Medicine, Columbus, Ohio; # University of Saskatchewan, Department of Emergency Medicine, Saskatchewan, Canada

## Abstract

**Introduction:**

Modern learners have immediate, unlimited access to a wide variety of online resources**.** To appeal to this current generation of learners, educators must embrace the use of technology. However, educators must balance newer, novel technologies with traditional methods to achieve the best learning outcomes. Therefore, we aimed to review several papers useful for faculty members wishing to incorporate technology into instructional design.

**Methods:**

We identified a broad list of papers relevant to teaching and learning with technology within the online discussions of the Academic Life in Emergency Medicine (ALiEM) Faculty Incubator. This list was augmented with suggestions by a guest expert (BT) and an open call on Twitter (tagged with the #meded and #FOAMed hashtags) yielding 24 papers. We then conducted a modified three-round Delphi process within the authorship group, including junior and senior faculty members, to identify the most impactful papers.

**Results:**

We pared the list of 24 papers to five that were most highly rated. Two were research papers and three were commentaries or editorials. The authorship group reviewed and summarized these papers with specific consideration to their value to junior educators and faculty developers.

**Conclusion:**

This is a key reading list for junior faculty members and faculty developers interested in teaching with technology. The commentary contextualizes the importance of these papers for medical educators, to optimize use of technology in their teaching or incorporate into faculty development.

## INTRODUCTION

Technology is changing the field of medical education.^1^ The acquisition of knowledge was previously limited by one’s access to experts, instructors or textbooks, but now learners have access to resources from around the globe. Learners can choose from digital textbooks, open-access journals, online encyclopedias, podcasts, blogs, free open-access medical education (FOAM), massive open online courses (MOOCs), the Khan academy, and TED talks.^2^–^7^

Educators have an ever-increasing variety of technologies to enhance their instructional design. They can deliver content synchronously or asynchronously (podcasts, vodcasts, blogs); and learners can be present in-person or virtually, creating the potential for online medical education to reach large audiences. Resources created by other educators can be leveraged on learning management systems (LMS) or used while implementing blended learning. Synchronous teaching sessions can be enhanced with simulation, videos, audience response systems, games, and live Twitter feeds.^8^–^10^

While such innovation in medical education has not traditionally been rewarded, promotion and tenure (P&T) committees have begun to consider social media and online metrics as evidence of scholarly merit in making promotional decisions.^11^

The Academic Life in Emergency Medicine (ALiEM) Faculty Incubator is an online faculty development initiative created to train early-career educators to teach in the 21st century. Rather than encouraging the use of technology for novelty’s sake, educators in the ALiEM Faculty Incubator aim at helping participants to understand educational theory, research, curricular design, and program evaluation so that they can maximize use of the most effective instructional design for meeting their teaching objectives. This integrative, narrative review was written to share key insights from the Incubator by highlighting the most important literature on technology in medical education for junior educators and faculty developers.

## METHODS

In the fifth month of the ALiEM Faculty Incubator (July 2016), we discussed teaching and learning with technology and exchanged key literature that members felt were relevant to this topic. We monitored the proceedings of this community of junior and senior faculty members from July 1–31, 2016, and compiled the papers mentioned in these discussions.

To ensure that we had a broad compendium of articles, we augmented our collection with papers suggested by the month’s guest expert (BT) and an open call for additional papers using Twitter with the hashtags #MedEd and #FOAMed. We subsequently conducted a three-round modified Delphi process to select papers relevant for faculty members on the month’s topic, based on the opinion of the selected panel members.^12^–^17^ Our selection panel included both novices (i.e., junior faculty members participating in the Faculty Incubator) and more experienced experts and educators (i.e., experienced clinician educators, all of whom have published >10 peer-reviewed publications, who serve as mentors and facilitators of the ALiEM Faculty Incubator), including the guest expert (BT, invited based on his past publications in the subject of teaching with technology). By mixing the opinions of the two groups, we sought to ensure that we selected papers of use to a spectrum of educators at different stages of their careers.

## RESULTS

The ALiEM Faculty Incubator discussions and social media calls yielded a total of 24 articles. The three-round voting procedure allowed our team to generate a rank-order listing of all these papers in order of importance for faculty members. The citations and ratings are listed in Table 1.

## DISCUSSION

The following is an annotated bibliography of the top papers, as determined by the modified Delphi process. The accompanying commentaries explain these papers’ relevance to faculty members using these articles for personal or other faculty development.

### 1. Roland D, Brazil V. Top 10 ways to reconcile social media and ‘traditional’ education in emergency care. *Emerg Med J.* 2015;32(10):819–22.^18^

#### Summary

New concepts and cultures within medical education make it difficult for educators to combine “technology-enhanced” education with traditional methods. This paper provides tips for reconciling the use of social media with more traditional education formats. The authors suggest that the principles for effectively using social media or podcasts in education are no different than other approaches: educators should provide clear learning objectives, assess learners, and evaluate teaching. Social media is one instructional strategy in education but cannot replace an entire curriculum.

#### Relevance to Junior Faculty Members

The paper provides valuable advice for junior faculty who are incorporating social media into their educational program. Junior faculty members should realize that newer, “technology-enhanced” methods of instruction are not automatically more effective. Many technologies are revisions of traditional methods and therefore must incorporate sound educational principles. For maximum efficacy, teaching with social media should use traditional educational objectives and program evaluation. Effective podcasting principles are similar to principles for an effective lecture. Curricula that incorporate social media should ensure that learners have study plans that include a wide range of topics, avoiding overemphasis of the popular topics at the expense of common, less-exciting subjects.^19^

Faculty members must help learners to consume FOAM in a manner similar to other medical literature. It may be valuable to assist learners in appraising resources or pre-selecting articles. In addition to serving as a method of receiving information, the authors highlight that social media may also have value as an outlet for reflection, feedback, and scholarly dissemination. Junior faculty should encourage learners to engage with social media and model examples of professionalism and confidentiality, as well as examples of quality academic scholarship.^20^

#### Considerations for Faculty Developers

Faculty developers should consider expanding their mentorship of junior faculty members to include reflection on the faculty members’ social media presence. Mentorship can be provided on professionalism and the effectiveness of educational programs. Faculty development programs may include instruction on how to expand faculty members’ social media knowledge and skills. Social media may also be used directly as a medium for faculty development. Social media allows for the distribution of faculty development content as well as the formation of virtual communities of practice.

### 2. Mehta NB, Hull AL, Young JB, Stroller JK. Just imagine: new paradigms for medical education. *Acad Med.* 2013;88(10):1418–23.^21^

#### Summary

Disruptive innovations are radical paradigms that offer a simpler, more convenient, customizable, or cheaper solution to a problem and may provide solutions to many of the current medical education system issues. The authors propose that MOOCs and digital badges may disrupt the educational system. MOOCs are repositories of online content that can reach large audiences across institutional boundaries. They can share large amounts of content at a low cost and would be capable of expanding and standardizing the delivery of high-quality education materials. Digital badges are digitally encoded elements that students can earn to reflect mastery of skills or specific achievements, in place of traditional grades. The authors’ vision for undergraduate and graduate medical education would use a central online collaborative learning environment and award transferable digital badges for competency-based assessments and advancement. The authors propose that such a system would facilitate interdisciplinary medical education, lifelong learning skills, and customization of learning outcomes. With less time devoted to didactic teaching, faculty could focus on higher level small-group discussions, observed assessments with formative feedback, and the verification of competency.

#### Relevance to Junior Faculty Members

Junior faculty may fail to consider how their work fits into the bigger picture, preventing meaningful change in the field. This editorial allows those junior faculty to see how work on disruptive innovations such as FOAM, MOOCs, digital badges, blended learning methodologies, and assessment methods could fit into the larger narrative of medical education evolution. While the discussion is idealistic, it may inspire junior faculty by introducing them to new, relatively uncharted fields within medical education.

#### Considerations for Faculty Developers

The paper examines the larger scale challenges in medical education. The authors predict that disruptive innovations and technology will play an important role in future changes in medical education, especially with respect to competency-based medical education. The paper provides a rationale for embracing change and potentially disruptive innovations. Operationalization of digital badges is supported by the growing focus within the medical education literature regarding Entrustable Professional Activities (EPAs) and Statements of Awarded Responsibility (STARs).^22^ Faculty developers should be familiar with these concepts in order to inform and inspire junior educators.

### 3. Thoma B, Chan TM, Paterson QS, Milne WK, Sanders JL, Lin M. Emergency medicine and critical care blogs and podcasts: establishing an international consensus on quality. *Ann Emerg Med.* 2015;66(4):396–402.e4.^23^

#### Summary

The use of FOAM has rapidly increased in popularity among learners. Despite this, the academic community has been hesitant to fully endorse these educational materials because of uncertainty regarding their quality. This paper is the first collaborative effort by experts to develop consensus regarding quality of online medical education resources. The authors used a modified Delphi of expert emergency medicine and critical care bloggers and podcasters to determine the relative importance of each quality indicator for blogs and podcasts, from a previously defined list of 151 quality indicators pertaining to credibility, content, and design.^24^ The authors invited expert participants (22 podcasters and 24 bloggers) to participate in the surveys, based on their position as lead editor(s) of one of the highest rated emergency medicine or critical care blogs/podcasts as determined by the Social Media Index.^25^ The experts reached greater than 70% consensus for 85 quality indicators (5 for blogs only; 41 for podcasts; 39 of these for both), with greater than 90% consensus for 31 of these (5 for bloggers; 17 for podcasters; 9 for both).

#### Relevance to Junior Faculty Members

This resource provides a method to assess the quality of non-traditional educational resources, which are increasingly used by learners. While the list is not exhaustive, these quality indicators can assist learners and educators in evaluating online resource quality. Junior faculty educators may consider these indicators when deciding upon FOAM resources for their learners and/or curricula. Furthermore, these indicators may be useful to junior faculty members who wish to develop quality FOAM.

#### Considerations for Faculty Developers

As P&T committees begin considering FOAM in an educator’s portfolio of scholarly activity,^11^ faculty developers should be familiar with potential metrics for assessing FOAM quality. This paper is one of a few derivation and Delphi studies proposing quality indicators for FOAM resources.^20^, ^26^–^28^ The paper may also foster a discussion about the lack of critical appraisal for other traditional secondary resources such as textbooks or narrative reviews.

### 4. Sherbino J, Arora VM, Van Melle E, Rogers R, Frank JR, Holmboe ES. Criteria for social media-based scholarship in health professions education. *Postgrad Med J.* 2015;91(1080):551–5.^20^

#### Summary

Social media has rapidly emerged as a tool for disseminating medical innovations and education. Clinician educators engage in scholarship as part of their core mission.^29^ However, traditional metrics for evaluating scholarly work are not readily applicable to social media-based scholarship. Furthermore, there are no publications identifying evaluation criteria or a formal definition for social media-based scholarship in medical education. While Thoma, et al. focused on quality metrics for blogs and podcasts, Sherbino et al. seeks to define criteria for them as scholarship.^20^,^23^

Fifty-two health professions educators from 20 organizations in four countries reviewed various themes in medical education scholarship that had been previously identified by an expert working group. The group unanimously agreed on four key features of social media-based scholarship: 1) it must be original (i.e., cannot simply re-broadcast material created elsewhere); 2) it must advance the field by building on best practice, research, or theory; 3) it must be disseminated and archived; and 4) it must provide the education community with the opportunity to give transparent feedback that informs a wider discussion.

#### Relevance to Junior Faculty Members

Junior faculty members are under pressure to produce academic scholarship to further their careers and advance towards promotion and tenure. Traditionally, such scholarship is in the form of peer-reviewed research. At the same time, medical learners are increasingly using non-traditional sources for learning. This creates disconnect between the needs of the learner and educator. Junior faculty may be interested in becoming thought leaders in social media-based medical education but are held back by concerns about career advancement and academic recognition. By defining key qualities for this type of scholarship, junior faculty are provided with a framework to guide their scholarly efforts in these areas. As further consensus is achieved, social media-based scholarship in health professions education will become a more defined part of academic advancement and contribution for clinician educators.

#### Considerations for Faculty Developers

Scholarship has and will continue to evolve. Social media-based resources have proven to be robust and durable ways to disseminate ideas that meet many of the traditional definitions of scholarship. Traditional, pre-publication peer review is a notable exception to this, however. Rigid adherence to such traditional paradigms is likely to limit faculty creativity and institutional flexibility. Faculty developers should consider the emerging role of social media as a form of scholarship if they wish to keep ahead of competing institutions and recruit tech-savvy educators and innovators. This paper provides a framework for the potential recognition of this work. This paper also is relevant to faculty who serve on their institutions’ P&T committees. The metrics identified by this paper will help guide the acceptance and assessment of digital scholarship to be used in making promotion decisions.

### 5. Toohey SL, Wray A, Wiechmann W, Lin M, Boysen-Osborn M. Ten tips for engaging the millennial learner and moving an emergency medicine residency curriculum into the 21st century. *West J Emerg Med.* 2016;17(3):337–43.^8^

#### Summary

This review article provides tips for engaging the millennial learner by optimizing the use of technology in resident education. The authors provide multiple suggestions for “flipping the classroom,” including the importance of providing well-vetted, learner-responsible content (i.e., pre-class resources); maintaining active learner engagement through the use of team-based or problem-based learning; and using existing resources (e.g. MedEdPORTAL, *Journal of Education and Teaching in Emergency Medicine*, CORD Teaching Cases) when appropriate. When lectures are used, the authors suggest pre-reading, short lecture sessions, and active learning techniques (e.g. pause procedures, simulation, small-group discussions, audience response systems). Finally, the authors discuss optimizing the use of technology by having a central, cohesive repository for all residency information (e.g. learning management system); using technology to provide more effective feedback; and providing frequent learning opportunities through residency-run blogs and automated teaching pearls.

#### Relevance to Junior Faculty Members

Toohey et al. provide a number of valuable tips for the junior faculty member. With increasing emphasis on learner-centered education and the incorporation of online media into resident education, it is important to use these resources effectively. The authors emphasize active learning strategies supported by effective pre-reading when preparing didactics. Active learning can be achieved through numerous strategies including audience response systems, team-based learning, problem-based learning, and simulation.^9^ When supervising residents on shift, the authors recommend rapid, real-time, formative feedback to avoid recall bias, enhance its impact on practice, and avoid surprising biannual summative assessments. Examples of technologically-enhanced, real-time feedback software include Instant Eval, New Innovations, MedHub, and MyEvaluations.com. When supervising resuscitations or procedures, optical head-mounted displays (e.g., Google Glass™, GoPro™ cameras) or video recording can further reduce recall bias and allow for the supervisor to focus on the patient, while allowing for valuable feedback to be provided later.

#### Considerations for Faculty Developers

The article provides strategies for incorporating technology into a residency curriculum. Faculty development is critical to the success of any educational program. This article could act as pre-reading for faculty development seminars or be added to a reading list for junior faculty members. The article specifically focuses on effective teaching strategies, using technology to organize and manage a residency curriculum, and using technology for providing learners with feedback.

## LIMITATIONS

First, the selection of articles for the modified Delphi process did not incorporate an exhaustive, systematic search of the literature. Rather, the knowledge of experts in the field was combined with crowd-sourced feedback from the online community to generate the list of articles that were evaluated. We believe that this process was able to identify some of the key papers on the topic of interest while keeping the list a manageable length for the participants in the modified Delphi process. However, it is possible that key articles could have been missed or excluded. Furthermore, the authors only considered journal articles, rather than including all sources on the subject of teaching with technology, such as blogs and podcasts.

Second, in a pure application of the Delphi technique all participants would be experts in faculty development. We declined this approach, opting instead to recognize the expertise of a range of faculty members. By incorporating junior faculty, we felt that we were better able to determine what is most relevant to them.

Third, the inclusion of authors within the field may have introduced bias in the selection of the articles. Being experts in the subject, the senior faculty were authors of many of the papers considered in the Delphi process. This limitation was balanced with the use of junior faculty who have not authored within the field and may have benefited article selection by accessing the expertise of these same authors.

Finally, given that all of the participants in the modified Delphi process were involved in the Faculty Incubator in some way, it is likely that they were not a representative sample of academic emergency medicine clinicians.

## CONCLUSION

This paper describes some key papers that may be useful to junior faculty members and faculty developers interested in teaching with technology. We believe that it will be helpful for clinician educators who seek to use technology effectively as the field of medical education continues to evolve at a rapid pace.

## Figures and Tables

**Table:** The complete list of educational scholarship literature involving social media and other online methods to enhance medical education, which were collected by the authorship team.

Citation	Round 1 initial mean scores (SD) max score 7	Round 2 % of raters that endorsed this paper	Round 3 % of raters that endorsed paper in last round	Top 5 papers
Roland D, Brazil V. Top 10 ways to reconcile social media and ‘traditional’ education in emergency care. *Emerg Med J*. 2015;32(10):819–22.^18^	6.3 (0.8)	100%	100%	1
Mehta NB, Hull AL, Young JB, Stroller JK. Just imagine: new paradigms for medical education. *Acad Med*. 2013;88(10):1418–23.^21^	5.5 (0.5)	87.5%	100%	2
Thoma B, Chan TM, Paterson QS, Milne WK, Sanders JL, Lin M. Emergency medicine and critical care blogs and podcasts: establishing an international consensus on quality. *Ann Emerg Med*. 2015;66(4):396–402.e4.^23^	5.2 (1.2)	100%	87.5%	3
Sherbino J, Arora VM, Van Melle E, Rogers R, Frank JR, Holmboe ES. Criteria for social media-based scholarship in health professions education. *Postgrad Med J*. 2015;91(1080):551–5.^20^	5.6 (0.9)	100%	87.5 %	4
Toohey SL, Wray A, Wiechmann W, Lin M, Boysen-Osborn M. Ten tips for engaging the millennial learner and moving an emergency medicine residency curriculum into the 21st century. *West J Emerg Med*. 2016;17(3):337–43.^8^	6.1 (0.8)	100%	87.5%	5
Chan TM, Grock A, Paddock M, Kulasegaram K, Yarris LM, Lin M. Examining reliability and validity of an online score (ALiEM AIR) for rating free open access medical education resources. *Ann Emerg Med.* 2016. [Epub ahead of print]^27^	5.7 (1.5)	37.5%	0%	
Bullock A and Webb K. Technology in postgraduate medical education: A dynamic influence on learning. *Postgrad Med J*. 2015;91(1081):646–50.^30^	5.7 (0.9)	25%	0%	
Sandars J, Patel RS, Goh PS. The importance of educational theories for facilitating learning when using technology in medical education. *Med Teach*. 2015;37(11):1039–42.^31^	5.5 (1.4)	62.5%	12.5%	
Scott KR, Hsu CH, Johnson NJ, Mamtani M, Conlon LW, DeRoos FJ. Integration of social media in emergency medicine residency curriculum. *Ann Emerg Med*. 2014;64(4):396–404.^32^	5.5 (1.2)	62.5%	0%	
Prober CG and Khan S. Medical education reimagined: a call to action. *Acad Med.* 2013;88(10):1407–10.^3^	5.0 (2.0)	37.5%	0%	
Stuntz R and Clontz R. An evaluation of emergency medicine core content covered by free open access medical education resources. *Ann Emerg Med.* 2016;67(5):649–653.e2.^19^	5.0 (1.2)	62.5%	12.5%	
Flynn L, Jalali A, Moreau KA. Learning theory and its application to the use of social media in medical education. *Postgrad Med J.* 2015;91(1080):556–60.^33^	4.9 (1.5)	62.5%	0%	
Hillman T and Sherbino J. Social media in medical education: a new pedagogical paradigm? *Postgrad Med J.* 2015;91(1080):544–5.^34^	4.9 (1.3)	0%	0%	
Cook DA, Hamstra SJ, Brydges R, et al. Comparative effectiveness of instructional design features in simulation-based education: systematic review and meta-analysis. *Med Teach.* 2013;35(1):e867–98.^35^	4.7 (1.8)	12.5%	0%	
Cook DA, Hatala R, Brydges R, et al. Technology-enhanced simulation for health professions education: a systematic review and meta-analysis. *JAMA*. 2011;306(9):978–88.^36^	4.5 (1.6)	12.5%	0%	
Chan TM, Thoma B, Krishnan K, et al. Derivation of two critical appraisal scores for trainees to evaluate online educational resources: A METRIQ study. *West J Emerg Med.* 2016;17(5):574–84.^28^	4.5 (1.4)	0%	0%	
Chan TM, Thoma B, Radecki R, et al. Ten steps for setting up an online journal club. *J Contin Educ Health Prof*. 2015;35(2):148–54.^37^	4.4 (1.3)	0%	0%	
Nickson CP and Cadogan MD. Free open access medical education (FOAM) for the emergency physician. *Emerg Med Australas.* 2014;26(1):76–83.^38^	4.1 (1.4)	12.5%	0%	
Mallin M, Schlein S, Doctor S, Stroud S, Dawson M, Fix M. A survey of the current utilization of asynchronous education among emergency medicine residents in the United States. *Acad Med.* 2014;89(4):598–601.^39^	3.8 (1.2)	0%	0%	
Bennett S, Maton K, Kervin L. The ‘digital natives’ debate: a critical review of the evidence. *Br J Educ Tech*. 2008;39(5):775–786.^40^	3.8 (0.9)	0%	0%	
Thoma B, Chan T, Desouza N, Lin M. Implementing peer review at an emergency medicine blog: bridging the gap between educators and clinical experts. *CJEM.* 2015 Mar;17(2):188–91.^41^	3.5 (0.8)	0%	12.5%	
Gooi AC. Is the textbook dead? Examining the technologies used by medical students to learn. *MedEdPublish*. 2014;3:5.^42^	3.3 (1.1)	0%	0%	
Desai B. A novel use of Twitter to provide feedback and evaluations. *Clin Teach*. 2014;11(2):141–5.^43^	3.0 (1.0)	0%	0%	
Hiltz SR. Impacts of college-level courses via asynchronous learning networks: some preliminary results. *JALN.* 1997; 1(2).^44^	2.1 (1.0)	0%	0%	
